# Exosomal microRNA/miRNA Dysregulation in Respiratory Diseases: From *Mycoplasma*-Induced Respiratory Disease to COVID-19 and Beyond

**DOI:** 10.3390/cells12192421

**Published:** 2023-10-09

**Authors:** Yingjie Wang, Mengyun Zou, Yabo Zhao, Md. Ahsanul Kabir, Xiuli Peng

**Affiliations:** Key Laboratory of Agricultural Animal Genetics, Breeding and Reproduction, Ministry of Education, College of Animal Science and Technology and College of Veterinary Medicine, Huazhong Agricultural University, Wuhan 430070, China; wang985@webmail.hzau.edu.cn (Y.W.); zoumengyun@webmail.hzau.edu.cn (M.Z.); yabozhao@mail.tsinghua.edu.cn (Y.Z.); kabir@blri.gov.bd (M.A.K.)

**Keywords:** *Mycoplasma*, miRNAs, respiratory disease, therapy

## Abstract

Respiratory diseases represent a significant economic and health burden worldwide, affecting millions of individuals each year in both human and animal populations. MicroRNAs (miRNAs) play crucial roles in gene expression regulation and are involved in various physiological and pathological processes. Exosomal miRNAs and cellular miRNAs have been identified as key regulators of several immune respiratory diseases, such as chronic respiratory diseases (CRD) caused by *Mycoplasma gallisepticum* (MG), *Mycoplasma pneumoniae* pneumonia (MMP) caused by the bacterium *Mycoplasma pneumoniae*, coronavirus disease 2019 (COVID-19), chronic obstructive pulmonary disease (COPD), asthma, and acute lung injury/acute respiratory distress syndrome (ALI/ARDS). Consequently, miRNAs seem to have the potential to serve as diagnostic biomarkers and therapeutic targets in respiratory diseases. In this review, we summarize the current understanding of the functional roles of miRNAs in the above several respiratory diseases and discuss the potential use of miRNAs as stable diagnostic biomarkers and therapeutic targets for several immune respiratory diseases, focusing on the identification of differentially expressed miRNAs and their targeting of various signaling pathways implicated in disease pathogenesis. Despite the progress made, unanswered questions and future research directions are discussed to facilitate personalized and targeted therapies for patients with these debilitating conditions.

## 1. Introduction

MicroRNAs (miRNAs) are small non-coding RNA molecules that are crucial regulators of gene expression through their ability to bind messenger RNA (mRNA) transcripts, leading to mRNA degradation or translational inhibition [1,2]. Dysregulation of miRNA expression is associated with altered cellular processes in pathological or abnormal physiological conditions, which can either promote or inhibit cellular processes depending on the specific miRNA involved [3]. Thus, a comprehensive understanding of the role of miRNAs in cellular signaling pathways and their dysregulation in pathological conditions is necessary to identify potential therapeutic targets.

It is important to note that the dysregulation of miRNA expression can be detected not only within cells, but also in other biological fluids [4]. One such fluid is exosomes, which are small membrane-bound vesicles released by cells and found in all biological fluids. These vesicles are essential in intercellular communication by delivering diverse biomolecules, including miRNAs, to recipient cells [5]. Due to their relative stability, exosomal miRNAs are believed to be crucial regulators of various biological processes and have potential therapeutic applications in numerous diseases [6].

Respiratory illnesses pose a significant economic and health burden worldwide, affecting millions of individuals annually in both human and animal populations [7,8]. Recent studies indicate that both exosomal miRNAs and cellular miRNAs play crucial roles in the pathogenesis and progression of numerous respiratory diseases, including chronic respiratory diseases (CRD) resulting from *Mycoplasma gallisepticum* (MG) infections, *Mycoplasma pneumoniae* pneumonia (MMP) caused by the bacterium *Mycoplasma pneumoniae* (MP), COVID-19, chronic obstructive pulmonary disease (COPD), asthma, and acute lung injury/acute respiratory distress syndrome (ALI/ARDS). The immune response is a critical component in the pathogenesis of these diseases [9,10,11,12,13,14].

The involvement of numerous miRNAs in respiratory disease development and progression has been well established; however, identifying appropriate miRNAs for therapeutic purposes is complicated by the fact that a single miRNA can target numerous genes and multiple miRNAs may effectively treat the same respiratory disease but exert diverse or contradictory effects by targeting distinct or overlapping gene networks [15,16]. Thus, the selection of miRNAs for therapeutic applications and the identification of optimal target genes pose a multifaceted challenge.

To tackle this complex issue, this review paper presents a comprehensive summary of all the miRNAs that have been identified in association with *Mycoplasma gallisepticum* (MG)-induced chronic respiratory diseases (CRD), along with information on their target genes. Additionally, a network diagram is presented to illustrate the targets and action pathways of MG-associated miRNAs, which can aid in the development of effective therapeutic strategies for CRD. Moreover, this review also provides an overview of the functional roles of miRNAs in other respiratory diseases, including *Mycoplasma pneumoniae* pneumonia (MMP), COVID-19, chronic obstructive pulmonary disease (COPD), asthma, and acute lung injury/acute respiratory distress syndrome (ALI/ARDS). The ultimate goal is to offer a comprehensive understanding of the potential for miRNAs as diagnostic and therapeutic targets in respiratory diseases.

## 2. Pathophysiology Roles of miRNA in *Mycoplasma gallisepticum*-Induced Respiratory Diseases

Respiratory illnesses caused by *Mycoplasma* infections have become a significant public health concern globally, affecting millions of individuals each year and imposing substantial economic and health burdens [17,18,19]. Among these are chronic respiratory diseases (CRD) caused by *Mycoplasma gallisepticum* (MG). In this section, we provide a comprehensive overview of the involvement of miRNAs in MG-induced CR, including their targets, potential use as diagnostic and therapeutic tools, and functional roles in regulating immune responses during respiratory infections. Moreover, we emphasize the importance of exosomal miRNAs in the diagnosis, prognosis, and treatment of respiratory diseases.

### 2.1. miRNAs in CRD

**Figure 1:**
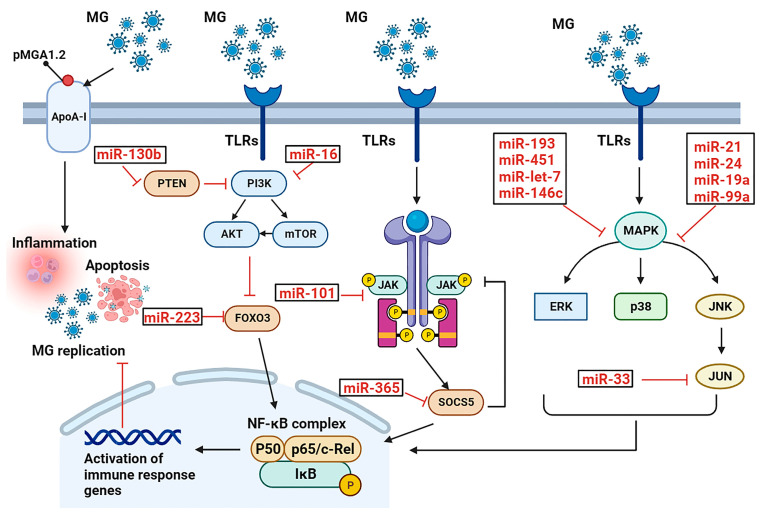
Networks of miRNAs and target genes in MG infection. Differently expressed miRNAs are denoted with red. During MG infection, miRNAs are involved in the regulation of JAK/STAT, JNK, TLRs/MyD88/NF-κB, PI3K-Akt, and MAPK pathways. In addition, these molecules can regulate cell proliferation, cell cycle progression, cellular inflammation, and apoptosis involved in the MG infection process.

In a previous study, deep sequencing was utilized to preidentify miRNAs associated with MG infection in chicken lungs at 3 and 10 days post-infection. A total of 45 and 68 differentially expressed miRNAs, which targeted 6290 and 7181 genes, were identified in lungs infected with MG at 3 and 10 days post-infection, respectively. These miRNAs were found to regulate a multitude of genes involved in various signaling pathways, including the MAP, TLR, Wn, and JAK/STAT pathways [10]. Additionally, the presence of certain miRNAs in exosomes and their ability to be exchanged between different cell types was observed. Exosomal miRNA sequencing data revealed that 30 miRNAs were significantly differentially expressed in the MG-infected group compared to the non-infected group [9]. Several of these miRNAs were selected for further study of their functions [9,20,21,22,23,24,25,26,27,28,29,30,31,32,33,34,35], and this review section summarizes our research on the roles of miRNAs and exosomal miRNAs in regulating signaling pathways during MG infection in chickens (Figure 1, Table 1).

Several miRNAs closely associated with MG infection were first identified by us. The JAK/STAT pathway is inhibited by MG through a negative feedback loop involving the downregulation of gga-miR-365-3p and SOCS5, which evades host immunity [20]. In addition, MG suppresses miR-223 expression in DF-1 cells, promoting self-infection [33].

The MAPK pathway is another key pathway in the MG infection process [9,10]. Upregulation of miR-142 and miR-24 alleviates inflammation by negatively regulating the MAPK signaling pathways and facilitates cell proliferation by inhibiting cell apoptosis to defend against MG infection [27,34]. Additionally, let-7d has been found to play a role in reducing the adhesion capacity of MG by suppressing the MAPK pathway through the activated target gene MKP1 [36].

It is interesting to note that miR-21 and miR-451, which are upregulated during MG infection, have opposite effects on cellular proliferation and apoptosis. miR-21 promotes cell proliferation and suppresses apoptosis, while miR-451 inhibits cellular proliferation and promotes apoptosis [9,28,29]. This suggests that MG may manipulate the host’s miRNA machinery to balance the cellular responses, allowing it to persist in the host without causing excessive damage. Targeting these miRNAs or their downstream targets may provide potential therapeutic strategies for the control of MG-induced respiratory diseases. Additionally, the regulation of the MAPK pathway and its associated signaling molecules by differentially expressed miRNAs during MG infection highlights the importance of this pathway in the host response to MG invasion and provides potential therapeutic targets for the treatment of MG-induced respiratory diseases.

miR-21 and miR-451 have been extensively researched in relation to Mycoplasma gallisepticum (MG) infection. In both MG-infected DF-1 cells and chicken embryonic lungs, the expression of both miRNAs is significantly upregulated. Bioinformatics analysis combined with luciferase reporter assays revealed that miR-21 directly targets MAP3K1, while miR-451 directly targets YWHAZ in the context of MG infection. MAP3K1 is a crucial member of the MAPK family, which plays a significant role in innate immune responses to pathogenic microorganism invasion. The inhibition of MAP3K1 by miR-21 activates the MAPK and NF-κB pathways, resulting in the generation of increased inflammatory cytokines. Moreover, miR-21 significantly promotes cell proliferation by increasing cell cycle progression and suppressing cell apoptosis to protect against MG infection. On the other hand, miR-451 inhibits the proliferation of DF-1 cells by preventing cell cycle progression and promoting cell apoptosis. YWHAZ regulates the MAPK p38 pathway in various diseases, and its targeting by miR-451 might regulate inflammatory cytokines by activating the MAPK p38 pathway to respond to MG infection.

Our study also highlights the crucial role of miRNAs in regulating various other pathways involved in MG infection, including the PI3K-Akt, MyD88/NF-κB, and JNK pathways. Specifically, the upregulation of miR-16-5p, miR-130b-3p, miR-19a, and miR-146c in both MG-infected chicken embryo lungs and DF-1 cells has been observed. miR-16-5p and miR-130b-3p target genes in the PI3K-Akt pathway, regulating cell proliferation and apoptosis during MG infection [31,35], while miR-19a and miR-146c upregulation promotes cell proliferation by activating the MyD88/NF-κB pathway while inhibiting apoptosis [30,32]. Interestingly, despite having opposite expression trends in MG-infected cells, miR-99 and miR-101-3p have similar regulatory effects on cell proliferation and cycle. The overexpression of miR-99a significantly inhibits DF-1 cellular proliferation by blocking the G1-to-S-phase transition, while the overexpression of miR-101-3p significantly hinders DF-1 cellular proliferation and cell cycle progression by suppressing EZH2 expression [22,26]. These findings shed new light on the mechanisms underlying MG pathogenesis and suggest that both upregulated and downregulated miRNAs can have comparable outcomes for DF-1 cell proliferation and cycle.

Additionally, the lncRNA targeting of miRNAs to regulate the JNK pathway during MG infection was also identified. Specifically, we identified lnc90386 as a sponge for miR-33-5p, which weakens its inhibitory effect on JNK1. By doing so, lnc90386 defends against MG infection by inhibiting inflammation and apoptosis in DF-1 cells [21]. This finding highlights the importance of the lncRNA-mediated regulation of miRNA function in the context of MG infection.

### 2.2. Exosomal miRNAs

Exosome-derived microRNAs in MG-infected chicken type II pneumocytes (CP-II) were investigated using sRNA sequencing analysis. Nine miRNAs, including miR-193a-3p, miR-33-5p, miR-460b-5p, miR-202-5p, miR-1784-5p, miR-6555-3p, miR-1783, miR-6696-3p, and miR-218-3p, were found to be upregulated, while 21 miRNAs, including miR-451, let-7d, and miR-133c-3p, were downregulated in exosomes compared to the control group. These differentially expressed miRNAs are mainly involved in MAPK, the cell cycle, apoptosis, and Toll-like receptor signals, consistent with previous miRNA sequencing results [9]. Notably, exosomal miR-451 was significantly downregulated in the MG-infected group, despite its increased expression in MG-infected CP-II cells, indicating the selective unloading of miR-451 in MG-infected exosomes. This selective packing of exosomal miRNAs has been observed in other studies, indicating that the miRNA content in exosomes differs significantly from that of parental cells [37,38]. Functional studies using DF-1 cells showed that miR-451-absent exosomes derived from MG-infected CP-II increased inflammatory cytokine production, including TNF-α and IL-1β. YWHAZ was identified as the direct target of miR-451, which regulated inflammatory cytokine production and cell proliferation upon MG infection [9].

In contrast to the selective unloading of miR-451 in exosomes, we conducted further investigation into the functions of two miRNAs that were found to be enriched in exosomes. miR-193a was observed to be enriched in exosomes derived from CP-II and was found to inhibit cell proliferation, promote apoptosis, and increase the secretion of inflammatory cytokines by targeting the RAS/ERK signaling pathway upon transportation to recipient cells [25]. Similarly, exosomal miR-181a-5p from CP-II activated the TLR2-mediated MyD88/NF-κB signaling pathways in recipient cells, thereby promoting the expression of pro-inflammatory cytokines and providing defense against MG infection [24].

In summary, it can be inferred that a multitude of miRNAs play a role in regulating the host immune response and influencing the pathogenesis of MG infection. The dysregulation of these miRNAs has a significant impact on crucial signaling pathways involved in the host immune response, such as the JAK/STAT, JNK, TLRs/MyD88/NF-κB, PI3K-Akt, and MAPK pathways. Furthermore, the dysregulation of miRNAs also affects the expression of cytokines and chemokines, which leads to inflammation and tissue damage. Therefore, targeting specific miRNAs could be a potential therapeutic strategy for the treatment of MG infection in poultry. However, further research is necessary to completely comprehend the precise mechanisms of miRNA regulation in MG infection and to develop effective miRNA-based therapies.

## 3. Pathophysiology Roles of miRNA in *Mycoplasma pneumoniae* Pneumonia

*Mycoplasma pneumoniae* pneumonia (MMP) is a bacterial pneumonia that is caused by *Mycoplasma pneumoniae* (MP) bacteria [39]. This type of pneumonia is frequently observed in children and young adults, particularly in settings such as schools and college dormitories, where large groups of people are in close proximity to each other [40]. Symptoms of MPP are similar to other forms of pneumonia and may include cough, fever, headache, muscle aches, and fatigue [41].

### 3.1. miRNAs in MMP

**Figure 2:**
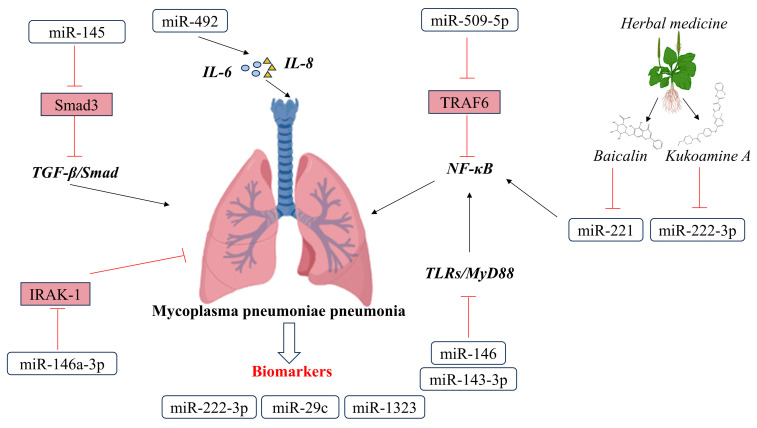
Networks of miRNAs and target genes in *Mycoplasma pneumoniae* (MP) infection.

Recent research has highlighted the significant role that miRNAs play in regulating *Mycoplasma pneumoniae* (MP) infection and the host immune response (Figure 2). For instance, miR-509-5p targets TRAF6 to negatively regulate the NF-κB pathway and modulate the inflammatory response in sheep infected with MP [42]. In mononuclear macrophages, miRNA-492 regulates the secretion of immune-inflammatory factors like IL-6 and IL-18, thereby contributing to the development of Mycoplasma pneumonia in children [43]. Additionally, miR-145 targets Smad3 to inhibit the TGF-β/Smad pathway, while miR-146a targets Toll-like receptors’ (TLRs) downstream signaling pathways, which are involved in regulating the intrinsic immune response [44]. The upregulation of miR-143-3p reduces alveolar epithelial cell apoptosis and mitigates lung inflammation in mice with mycoplasma pneumonia by inhibiting the TLR4/MyD88/NF-κB signaling pathway, and miR-146a-5p exhibits therapeutic potential in refractory MMP by decreasing the protein expression of ATP-binding cassette subfamily G member 1 (ABCG1) and interleukin 1 receptor-associated kinase 1 (IRAK-1) [45,46].

### 3.2. Possibilities of miRNA in the Treatment of Mycoplasma pneumoniae Pneumonia

The drug-mediated modulation of miRNA expression has emerged as a promising strategy in treating *Mycoplasma pneumoniae* pneumonia (MMP). Baicalin, a flavonoid compound found in *Scutellaria baicalensis*, has been shown to inhibit miR-221 expression and regulate the TLR4/NF-κB signaling pathway, thereby demonstrating therapeutic potential for lung injury induced by MP infection [47]. In MPP patients, miR-222-3p expression was found to be significantly upregulated compared to healthy individuals [48]. However, Kukoamine A (KuA), a natural compound isolated from the Chinese herb Cortex Lycii Radicis, has been found to attenuate the effects of the miR-222-3p/SOD2 axis, indicating its potential as a promising therapeutic strategy for MPP [49]. Moreover, in a lamp-induced MPP model, the upregulation of miR-222-3p or knockdown of TIMP3 has been shown to reverse the promotion of cell viability and the suppression of inflammation induced by GAS5 overexpression [50].

### 3.3. Possibilities of miRNA in the Diagnosis of Mycoplasma pneumoniae Pneumonia

Furthermore, recent studies have highlighted the potential of miRNAs as biomarkers and therapeutic targets for MMP. One study conducted on children with MPP found that miR-29c levels were significantly lower and sB7-H3 and IL-17 levels were higher during the acute phase compared to healthy children. However, during the recovery period, miR-29c levels increased, and sB7-H3 and IL-17 levels decreased. Furthermore, sB7-H3 levels were significantly higher in children with MPP accompanied by pleural effusion, and they correlated positively with the number of fever days. The study suggested that miR-29c and B7-H3 could be potential targets for the control of MPP and assessment of prognosis [51]. In another study, plasma samples of children with MPP identified miR-222-3p as a promising biomarker for diagnosis and prognosis [48]. Additionally, miR-1323 was found to have therapeutic potential as its overexpression reduced the expression of IL-6 in THP-1 cells, indicating its potential in the treatment of MPP [52]. Overall, these findings suggest that miRNAs could be promising targets in controlling MMP and assessing its prognosis.

## 4. Pathophysiology Roles of miRNAs in COVID-19

**Figure 3:**
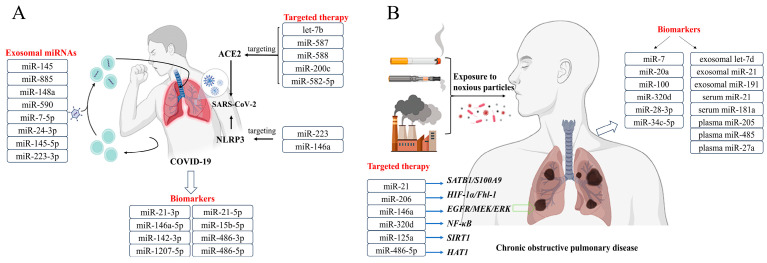
Multiple functions of miRNAs in COVID-19 (**A**) and COPD (**B**), including biomarkers, diagnostic markers, and targeted therapies.

The COVID-19 pandemic, caused by SARS-CoV-2, has had a significant impact on public health globally. It is believed that a dysregulated immune response, characterized by excessive pro-inflammatory cytokine production and immune cell death, contributes to the severity of the disease [53]. Recent studies have suggested that miRNAs may also play a role in the pathogenesis of COVID-19 [54]. In this section, we summarize the current understanding of miRNA dysregulation in COVID-19 (Figure 3A, Table 2).

### 4.1. miRNAs in COVID-19

One study reported that SARS-CoV-2 may act as an exogenous competing RNA, leading to the dysregulation of miR-1207-5p targets involved in uncontrolled inflammatory responses in COVID-19 [55]. In mouse models of SARS-CoV infection, miR-21-3p was found to be upregulated in lung tissue, suggesting a potential interaction between miR-21-3p and human coronavirus transcripts [56]. Other studies have identified differentially expressed miRNAs in COVID-19 patients, with miR-146a-5p, miR-21-5p, and miR-142-3p consistently downregulated in patients with moderate or severe disease, and miR-3605-3p upregulated [57]. miR-15b-5p, miR-486-3p, and miR-486-5p were overexpressed only in severely affected COVID-19 cases, while miR-181a-2-3p, miR-31-5p, and miR-99a-5p were only downregulated in this subtype of COVID-19 cases [57]. These miRNAs were found to be enriched in pathways related to inflammation, the antiviral immune response, Toll-like receptor (TLR) signaling, and IFN-related pathways.

### 4.2. Possibilities of miRNA in the Diagnosis of COVID-19

miR-146a-5p was identified as one of the most significant miRNAs affecting gene expression in the lungs of COVID-19 patients [58]. A reduction in miR-146a-5p levels was observed in the serum of non-responding COVID-19 patients after receiving Tocilizumab, and lower expression levels of this miRNA were associated with poorer outcomes [59]. Moreover, several miRNAs, including miR-21, miR-126, miR-155, miR-208a, and miR-221, were linked to clinical outcomes in COVID-19 patients. miR-21, which is involved in regulating inflammatory responses, was found to be upregulated in the peripheral blood of COVID-19 patients and positively correlated with disease severity [60]. On the other hand, miR-126, which plays a role in vascular endothelial function, was downregulated in the peripheral blood of COVID-19 patients and associated with severe disease. Increased expression of miR-155 was found in the peripheral blood of COVID-19 patients with mild symptoms, suggesting a potential protective role for this miRNA. Additionally, miR-208a was associated with myocardial injury in COVID-19 patients, demonstrating the potential usefulness of miRNAs as biomarkers for disease progression and severity [60].

### 4.3. Possibilities of miRNA in the Treatment of COVID-19

Apart from their potential as diagnostic and prognostic markers, miRNAs can also be targeted therapeutically in COVID-19. For instance, miR-200c and let-7b were found to regulate the expression of ACE2, which is the receptor for SARS-CoV-2. Inhibition of these miRNAs was shown to increase ACE2 expression and enhance viral entry into cells, while overexpression had the opposite effect [61,62]. Additionally, miR-588, miR-587, and miR-582-5p have been predicted to regulate ACE2 levels and potentially play a role in the progression of COVID-19, making them potential targets for the prevention of SARS-CoV-2 infection [68]. Similarly, miR-223 and miR-146a have been found to regulate the immune response to SARS-CoV-2 by targeting the NLRP3 inflammasome pathway. Inhibition of miR-223 and miR-146a has been shown to enhance the antiviral immune response, while overexpression had the opposite effect. Thus, targeting these miRNAs can be a potential therapeutic strategy for COVID-19 [63,64].

Exosomal miRNAs may play a role in the coagulation system and neurological symptoms observed in COVID-19 patients. Research has shown that miR-145 and miR-885, which are involved in regulating thrombosis, are reduced in exosomes from COVID-19 patients compared to healthy individuals. These miRNAs are also involved in the expression of von Willebrand factor, which is involved in blood coagulation and elevated in COVID-19 patients [65]. This suggests that exosomal miRNAs could be contributing to the increased risk of thrombotic complications in COVID-19 patients. Additionally, dysregulated miRNA expression in the central nervous system has been observed in response to viral infections, and a recent study found that miR-148a and miR-590 were significantly upregulated in the exosomes of COVID-19 patients with neurological symptoms. Furthermore, low levels of certain miRNAs, including miR-7-5p, miR-24-3p, miR-145-5p, and miR-223-3p, in serum exosomes have been associated with high mortality rates in elderly and comorbid COVID-19 patients. These findings suggest that exosomal miRNAs could be targeted for therapeutic purposes in COVID-19 treatment [67].

In summary, miRNAs and exosomal miRNAs are essential components in the immune response to SARS-CoV-2 infection, and their dysregulation may contribute to the severity of the disease and the development of specific clinical manifestations in COVID-19 patients. Investigating miRNAs and exosomal miRNAs may provide valuable insights into the development of novel diagnostic and therapeutic options for COVID-19.

## 5. Pathophysiology Roles of miRNAs in COPD

Chronic obstructive pulmonary disease (COPD) is a chronic lung disease that causes breathing difficulties and is one of the leading causes of death worldwide [69]. In recent years, microRNAs have been extensively studied in COPD and may contribute to the varying symptoms and characteristics of the disease [66] (Figure 3B, Table 3).

### 5.1. miRNAs in COPD

A study was conducted to analyze miRNAs in patients with COPD who were treated with inhaled corticosteroids (ICS). It was found that miR-320d was significantly increased in bronchial biopsies following ICS treatment and was able to suppress the production of inflammatory cytokines by regulating the activity of NF-κB [80]. If future studies confirm the relationship between miR-320d and ICS treatment, miR-320d mimics could potentially be explored as a new treatment option or complement to ICS for COPD patients.

Dysregulation of exosomal miRNAs has been observed in COPD, with high levels of exosomal miR-21 detected in the serum of smokers and COPD patients. A functional study revealed that exosomal miR-21 targets pVHL, which regulates myofibroblast differentiation through HIF-1α signaling, suggesting that exosomal miR-21 may have diagnostic and therapeutic potential for COPD [81]. Other miRNAs enriched in cigarette smoke-released exosomes, including let-7d and miR-191, have been shown to potentially contribute to the pathogenesis of COPD by affecting the clearance of apoptotic cells by specialized macrophages [70].

### 5.2. Possibilities of miRNA in the Diagnosis of COPD

Biofluids such as serum, sputum, and exhaled breath condensates have been used to identify stable and easily detectable miRNAs, which could serve as potential biomarkers in predicting the development of COPD in asymptomatic smokers [71]. Serum miR-21 and miR-181a levels and their ratio have been suggested as potential biomarkers in predicting the development of COPD in asymptomatic smokers [72]. Five miRNAs (including miR-100, miR-20a, miR-34c-5p, miR-28-3p, and miR-7) were differentially expressed between patients with COPD and controls, indicating their potential as biomarkers for COPD [82]. These miRNAs may also have a biological function in the pathogenesis of COPD. Sputum and exhaled breath condensates have been used as easily accessible body fluids for the detection of miRNAs [75,82]. O’Farrell et al. used EV–miRNAs for COPD and lung cancer screening. They isolated EVs from plasma samples of healthy non-smokers, healthy smokers, lung cancer patients with a smoking history, and stable COPD patients. They extracted RNA from EVs and analyzed miRNAs, identifying 15 differentially expressed miRNAs between lung cancer patients and healthy non-smokers, 26 downregulated miRNAs in lung cancer patients versus healthy smokers, and 14 significantly dysregulated miRNAs in lung cancer patients versus stable COPD patients. These findings underscore the potential of EV–miRNAs as specific signatures indicative of pathological states [76,83]. In summary, miRNAs in biofluids have great potential in developing minimally invasive diagnostic biomarkers for COPD.

### 5.3. Possibilities of miRNA in the Treatment of COPD

Moreover, miRNA-based therapies have emerged as a promising strategy to treat COPD. Apart from miR-320d [80] and exosomal miR-21 [81], which have potential therapeutic value, other miRNAs, such as miR-206 and miR-146a, have also been identified as potential targets for COPD therapy [84,85]. These miRNAs are involved in regulating various aspects of COPD pathogenesis, such as inflammation, oxidative stress, and tissue repair. Therefore, targeting these miRNAs could potentially alleviate COPD symptoms and improve lung function. Future research on the use of miRNA-based therapies for COPD treatment is warranted.

## 6. Pathophysiology Roles of miRNAs in ALI/ARDS

Acute lung injury (ALI) and acute respiratory distress syndrome (ARDS) are lung diseases that can result in significant morbidity and mortality. Ongoing research aims to develop molecular-based therapies and prognostic biomarkers to improve the clinical management of these conditions [73].

### miRNAs in ALI/ARDS

**Figure 4:**
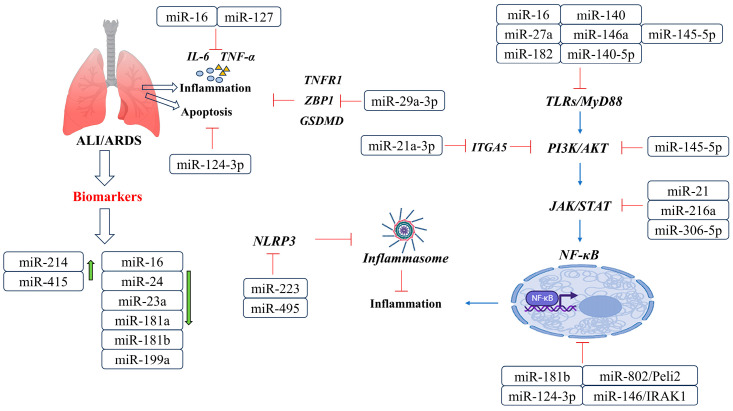
Networks of miRNAs and target genes in ALI/ARDS. During ALI/ARDS, miRNAs are involved in the regulation of JAK/STAT, TLRs/MyD88/NF-κB, PI3K-Akt, and NLRP3 pathways. In addition, these molecules can regulate cellular inflammation and apoptosis.

Studies have investigated the role of miRNAs in ALI/ARDS and have identified several differentially expressed miRNAs in these conditions (Figure 4, Table 4). For example, in an LPS-induced ALI mouse model, miR-214 and miR-415 were upregulated, while miR-16, miR-23a, miR-24, miR-181a, miR-181b, and miR-199a were downregulated. One study suggests that miR-16 may alleviate ALI by inhibiting LPS-induced IL-6 and TNF-a in cells [74]. Another study found that miR-29a-3p was significantly downregulated in ARDS patients and may play a role in regulating the inflammatory response by targeting TNFR1. The injection of miR-29a-3p agomir reduced alveolar epithelial cell PANoptosis in an ALI mouse model by downregulating ZBP1, GSDMD, caspase-3, caspase-8, and MLKL, leading to an improvement in lung injury [77]. These findings highlight the potential of miRNAs as therapeutic targets and biomarkers for ALI/ARDS, and further research is needed to fully understand their roles in the pathogenesis of these conditions.

More importantly, multiple research studies have found that miRNAs play a crucial role in the pathological process of ALI/ARDS through various signaling pathways, such as TLR4/NF-κB, JAK2/STAT3, PI3K/AKT, and NLRP3. The TLR4/NF-κB pathway is particularly important in regulating the inflammatory response in ALI/ARDS. Certain miRNAs, like miR-27a, miR-16, miR-182, miR-145-5p, miR-140, miR-140-5p, and miR-146a, have been shown to decrease TLR4 expression by targeting its 3′-UTR. This action reduces the release of pro-inflammatory cytokines and downstream TLR4/MyD88/NF-κB signaling pathways, ultimately suppressing the inflammatory response [78,79,86,87,103]. Moreover, miR-146b has been found to decrease lung inflammation and increase lung permeability by targeting IRAK1 and inhibiting NF-κB signaling [88]. On the other hand, miR-124-3p promotes macrophage apoptosis and plays a protective role in ARDS by targeting p65 [89]. The JAK2/STAT3 pathway is also a critical mediator of inflammation in ALI/ARDS. Studies have confirmed that LPS-induced ALI/ARDS inhibits miR-21 expression and activates JAK2/STAT3 and NF-κB signal transduction. The upregulation of miR-21 inhibits the JAK2/STAT3 pathway, thus reducing the infiltration of inflammatory cells in the lung tissue of LPS-induced ALI/ARDS mice [90]. Similarly, miR-216a can inhibit the JAK2/STAT3 pathway, thereby reducing cell apoptosis, autophagy, and the release of inflammatory factors, thus reducing LPS-induced ALI/ARDS [91]. Additionally, miR-30b-5p negatively regulates the JAK2/STAT3 pathway that mediates the inflammation of lung macrophages and inhibits the expression of ALI/ARDS inflammatory factors [92]. Furthermore, miR-127 has been shown to suppress lung inflammation by targeting macrophage CD64 [93].

The role of miRNAs in regulating ALI/ARDS also involves the PI3K/AKT pathway. For instance, miR-92a targets ITGA5 to inhibit the pathway, thereby improving endothelial cell barrier function and protecting alveolar vascular endothelial cells [94]. In addition, miR-21a-3p in stromal cell Telocytes is involved in regulating the PI3K (p110α)/Akt/mTOR pathway, which promotes lung tissue repair and angiogenesis, thus aiding in the recovery of ARDS in mouse models induced with LPS [95]. Furthermore, the NLRP3 pathway plays a crucial role in ALI/ARDS, and the overexpression of miR-495 and miR-223 inhibits NLRP3 activation, resulting in reduced inflammation and improved ALI/ARDS [96]. Additionally, targeting Peli2 with miR-802 improves lung injury induced by LPS [97].

It is important to note that some studies have produced conflicting results regarding the influence of specific miRNAs on ALI/ARDS. For instance, while miR-181b has been found to suppress the NF-κB signaling pathway by targeting importin-α3, a vital protein involved in the nuclear translocation of NF-κB, other research has detected an increase in miR-181b levels after LPS treatment, which activates the NF-κB signaling pathway by elevating p65 levels [98]. These inconsistencies underscore the intricate nature of miRNA–disease interactions and emphasize the necessity for additional investigations to fully comprehend the role of miRNAs in the pathogenesis of ALI/ARDS.

In summary, this section presents a broad overview of the contribution of miRNAs in the development of ALI/ARDS and highlights their effectiveness in mitigating inflammation through various pathways, as demonstrated by several studies. Additionally, specific miRNAs have exhibited potential as biomarkers for ALI/ARDS, indicating that regulating miRNA expression may offer a promising treatment strategy for lung injury in the future.

## 7. Pathophysiology Roles of miRNAs in Asthma

Asthma is a respiratory condition characterized by chronic inflammation and limited air flow [99]. The inflammation of the airways drives asthma symptoms by triggering various processes, such as mucus production, airway wall remodeling, and bronchial hyperresponsiveness (BHR), which causes smooth muscle cells to react to non-specific stimuli such as cold air [99].

### 7.1. miRNAs in Asthma

Recent research has identified several miRNAs that are involved in asthma pathogenesis and progression [100] (Table 5). For instance, miR-34a regulates the proliferation, migration, and apoptosis of airway smooth muscle cells and modulates airway inflammation by regulating cytokine expression, including IL-6 and IL-8 [102]. miR-26-5p regulates airway remodeling by regulating collagen gene expression [101], and miR-206 modulates airway inflammation by regulating the expression of the pro-inflammatory cytokine IL-25 [104]. miR-21 is a well-studied miRNA that is upregulated in asthmatic airways [105]. It promotes airway remodeling by regulating the expression of MMPs, which are enzymes that degrade the extracellular matrix [106]. Another miRNA implicated in asthma is miR-155 [107], which promotes the proliferation of Th cells through the downregulation of CTLA-4, thereby contributing to the development of allergic asthma [108]. Additionally, miR-155 is involved in the regulation of asthma by regulating the T(H)2 response via the transcription factor PU.1 [109]. Therefore, delivering miR-155-miRNAs via EVs may serve as a potential therapeutic strategy for AHR in asthma and requires further investigation.

### 7.2. Possibilities of miRNA in the Treatment of Asthma

miRNA-based therapies have emerged as a promising approach to treating asthma, given the significant role that miRNAs play in the pathogenesis and progression of the disease. One such therapy involves using miRNA mimics, which are synthetic RNA molecules that replicate the function of natural miRNA. These mimics are designed to target specific dysregulated miRNAs in asthma and restore their expression to normal levels. For example, in animal models of asthma, miR-146a mimics have demonstrated the ability to decrease airway inflammation and remodeling [122]. Similarly, the administration of let-7 mimics in the lungs of asthmatic mice has shown a reduction in IL-13 levels in tissues, BALF, and serum, resulting in a significant reduction in airway hyperresponsiveness to acetylcholine [110]. Another approach involves using anti-miRNA oligonucleotides, which are synthetic RNA molecules designed to inhibit specific miRNAs’ function. For instance, the use of anti-miR-21 oligonucleotides has been shown to decrease the upregulated miR-21 in asthma to normal levels, leading to a reduction in airway inflammation and remodeling [123].

### 7.3. Possibilities of miRNA in the Diagnosis of Asthma

miRNAs have been investigated as potential biomarkers for asthma, in addition to their use in miRNA-based therapies. Studies have shown that miR-1248, miR-155, 26a-1-3p, and miR-376a-3p are dysregulated in the serum and bronchoalveolar lavage fluid of patients with asthma [111,112,124]. A study conducted in Sweden analyzed the miRNA content of exosomes isolated from the BALF of asthma patients and found that the levels of 18 miRNAs were altered, with eight of them (let-7a, miRNA-21, miRNA-658, miRNA-24, miRNA-26a, miRNA-99a, miRNA-200c, and miRNA-1268) showing significant changes in expression [113]. A strong correlation was observed between the altered miRNA expression profile and the forced expiratory volume in 1 s (FEV1) in asthmatic patients [113]. miRNA-140-3p has also been found to play a significant role in airway smooth muscle cell hyperplasia, with its upregulation being demonstrated in the circulating exosomes of patients with severe asthma as compared to patients with mild-to-moderate asthma and healthy controls [125]. This suggests that miRNA-140-3p may serve as an important prognostic biomarker for severe asthma. Other miRNAs, such as members of the let-7 and miRNA-200 families, have also been shown to be dysregulated in exosomes isolated from BAL fluid in asthmatic patients [113,114,115,116,117]. These findings emphasize the potential usefulness of miRNAs as biomarkers for asthma and indicate that further research is needed to fully understand their roles in the pathogenesis and treatment of the disease.

## 8. Discussion and Conclusions

In this comprehensive study, we conducted an extensive comparative analysis of miRNA dysregulation in Mycoplasma-induced respiratory diseases and other prominent respiratory illnesses, encompassing COVID-19, COPD, asthma, and ALI/ARDS. Our overarching objective was to discern potential miRNAs with conserved roles across these diverse diseases, thus laying the groundwork for the repurposing of therapeutic strategies. Building upon our insights from Mycoplasma-induced respiratory diseases, we propose the exploration of therapeutic avenues applicable to a broader spectrum of respiratory disorders.

The significant impact of miRNAs on inflammation and immune responses in the context of MG infection suggests promising therapeutic potential across various respiratory conditions. Notably, miR-21’s capacity to combat MG infections aligns with its pivotal role in mitigating COVID-19, COPD, asthma, and ALI/ARDS [29,72,105,111,118]. Additionally, miR-21 has demonstrated its ability to ameliorate pulmonary fibrosis and inhibit lung cancer progression [119,120]. Conversely, the let-7 family of miRNAs appears to be associated with the exacerbation of lung diseases [36,68,121]. Therefore, manipulating miR-21 expression or let-7 inhibition could prove effective in treating lung diseases. Significantly, miRNAs exhibit distinct functions across different cell types. For instance, miR-223 promotes the infection process in respiratory cells while exerting anti-inflammatory effects in immune cells like macrophages [63,96,126]. Tailoring therapeutic strategies necessitates the consideration of cell and tissue specificity. Furthermore, our analysis of miRNA roles across various immune cell types led to the identification of promising miRNA co-targets, including PTEN, NLRP3, and MMPs [96,103,127,128,129]. Manipulating these targets, independent of the miRNA influence, presents an alternative therapeutic intervention for lung diseases.

Furthermore, lung diseases rarely occur in isolation; they often interact with one another. It would be highly informative to explore the significance of miRNAs in the complex relationships between various pulmonary conditions. (1) Shared Risk Factors and Common Pathways: Lung diseases, such as COPD and lung cancer, are frequently driven by common risk factors, primarily tobacco exposure. This shared etiology suggests that overlapping molecular pathways may be involved. Studies have indicated that miR-210 can be utilized for the early detection of both COPD and lung cancer [130,131]. Additionally, miRNA-33a-5p, 199a-5p, and 320a-3p levels were found to be elevated in both lung cancer and COPD patients compared to healthy controls [132]. An increase in miR-33a-5p may lead to the downregulation of SMAD Family Member 7 (SMAD7) and Zinc Finger Protein 281 (ZNF281), resulting in a loss of tumor suppressor ability that accelerates the progression of lung cancer and COPD [132,133]. (2) MiRNAs as Molecular Bridges: MiRNAs, small RNA molecules, post-transcriptionally regulate gene expression by binding to messenger RNAs (mRNAs). In the context of lung diseases, miRNAs can serve as molecular bridges connecting different conditions. For example, miR-15b, which promotes inflammation in COPD, might also be implicated in the development of lung cancer by fostering a pro-inflammatory microenvironment conducive to tumor growth [134]. (3) Disease Progression and Transitions: Lung diseases often progress over time, with one condition leading to or exacerbating another. miRNAs might play a crucial role in these disease transitions. For instance, in COVID-19 patients, the upregulation of the miR-200 family could contribute to lung inflammation. These miRNAs might then facilitate the transition to lung cancer by promoting genetic mutations or the growth of existing cancerous cells [135,136]. (4) Therapeutic Opportunities: Exploring the miRNA-mediated connections between lung diseases can open up new therapeutic avenues. miRNAs can be targeted or modulated to influence disease outcomes. Identifying miRNAs that are central to the interplay between different lung conditions could pave the way for innovative treatment strategies. For example, by targeting specific miRNAs (including miR-155, miR-197-3p, and miR-320a-3p) involved in inflammation common to both COPD and lung cancer, it might be possible to develop therapies with a broader impact [137,138,139]. In essence, miRNAs represent a molecular nexus in the complex web of interactions between different lung diseases. Investigating their roles in these relationships not only enhances our understanding of disease mechanisms but also offers exciting opportunities for the development of more targeted and effective therapeutic approaches and personalized medicine strategies.

Consistently dysregulated miRNAs across distinct respiratory diseases may serve as valuable diagnostic markers, beyond their therapeutic implications. The increasing significance of liquid biopsy, a non-invasive diagnostic tool, has brought attention to exosomal miRNAs (miRNAs enclosed in exosomes) in various diseases, including cancer and respiratory conditions. Exosomes are small vesicles secreted by cells containing bioactive molecules, including miRNAs, critical for intercellular communication. Exosomal miRNAs in liquid biopsy offer numerous advantages. (1) Non-Invasive Sampling: Exosomal miRNAs can be easily isolated from readily accessible bodily fluids such as blood, urine, and saliva. This non-invasive sampling method eliminates the need for invasive tissue biopsies, reducing patient discomfort and the risk of complications. (2) Convenience and Reproducibility: Collecting bodily fluids for liquid biopsy is a relatively straightforward and repeatable process, ensuring consistent and reliable results across multiple tests. (3) Stability and Protection: miRNAs enclosed within exosomes are shielded from degradation by RNases, extreme pH conditions, and other extracellular factors. This protection ensures the integrity and stability of miRNAs during sample collection, transportation, and storage, which is critical for accurate analysis. (4) Specificity: Exosomal miRNA profiles can be highly specific to particular diseases or conditions. Changes in the expression levels of specific miRNAs within exosomes can serve as unique biomarkers for different diseases, enabling precise diagnostics. (5) Sensitivity: Exosomal miRNAs offer remarkable sensitivity for disease detection. Even the slightest changes in miRNA expression can be detected, making them valuable for early disease diagnosis and disease progression monitoring. (6) Dynamic Information: Exosomal miRNAs can provide dynamic information about disease status and progression. Monitoring changes in miRNA expression over time can provide insights into treatment effectiveness and disease evolution. (7) Potential for Personalized Medicine: The unique miRNA signatures found in exosomes can help to customize treatments for individual patients, supporting the concept of personalized medicine, where therapies can be optimized based on the patient’s specific disease profile. (8) Therapeutic Potential: Beyond diagnostics, exosomal miRNAs have therapeutic potential. They can be engineered and utilized as therapeutic agents to modulate gene expression in recipient cells, providing novel approaches to treating diseases at the molecular level. Notably, miR-155, particularly within exosomes, emerges as a compelling candidate for non-invasive diagnostic tests, enabling early disease detection and progression monitoring. Elevated miR-155 levels across various lung diseases, including MMP, COVID-19, COPD, asthma, and ALI/ARDS, offer diagnostic opportunities, facilitating timely, personalized therapeutic interventions and enhancing disease management [107,109,138,140,141,142,143,144,145,146,147,148,149]. In summary, exosomal miRNAs in liquid biopsy have immense potential to revolutionize diagnostics and therapeutics. Their non-invasive nature and wealth of information make them a promising area of research in understanding and managing various diseases, including respiratory conditions, where they could aid in early detection, monitoring, and targeted therapy development.

However, it is crucial to acknowledge the challenges and limitations associated with miRNA-based therapies. The intricate interplay between unique pathogenic mechanisms, microenvironments, and varying cellular responses demands tailored approaches. While innovative methods like extracellular vesicle-based or nanomaterial-mediated miRNA delivery have emerged, achieving targeted delivery to specific cell types, particularly infected respiratory cells and immune cells, remains a formidable challenge. Addressing these complexities necessitates a comprehensive understanding of disease-specific molecular pathways and cellular microenvironments, coupled with the development of innovative delivery systems. In addition, miRNA expression is dynamic and subject to variations based on factors such as the disease stage, age, gender, and other clinical patient characteristics. This variability can complicate the establishment of a universal miRNA biomarker for a specific disease. Consequently, researchers often need to consider these variables when interpreting miRNA data and may opt for miRNA panels to enhance the diagnostic accuracy. Ethnic and population variability is another consideration, as miRNA expression can vary among different ethnic groups due to unique genetic and environmental factors. Therefore, miRNA-based biomarkers may require validation across diverse populations to ensure accuracy and reliability in various patient groups. Specific single-nucleotide polymorphisms (SNPs) can influence miRNA expression by affecting miRNA genes, target genes, or miRNA binding sites. Consequently, genetic variations among individuals should be taken into account when assessing miRNAs’ potential as biomarkers. The collection, storage, and processing of biological samples containing miRNAs can significantly impact the measurement reliability. Inconsistencies in these pre-analytical factors can introduce variability in miRNA data. Furthermore, miRNAs are part of complex gene regulatory networks, targeting multiple genes involved in different pathways. This complexity can make it challenging to pinpoint the precise role of a specific miRNA in disease pathogenesis. It is essential to note that the correlation between altered miRNA expression and disease does not necessarily imply causation. Functional studies are crucial to confirm the biological relevance of specific miRNAs in disease processes. Standardization is a critical concern as well. The lack of standardized protocols for miRNA analysis, including quantification methods and data normalization, can lead to result variability across different laboratories.

Given these limitations, researchers and clinicians must interpret miRNA data cautiously when considering them as potential biomarkers. While miRNAs offer great promise, their clinical application requires rigorous validation, considering various factors and complementing diagnostic approaches. Developing robust, standardized methods for miRNA analysis and interpretation is also imperative to unlock their full potential in clinical settings.

**Figure 5:**
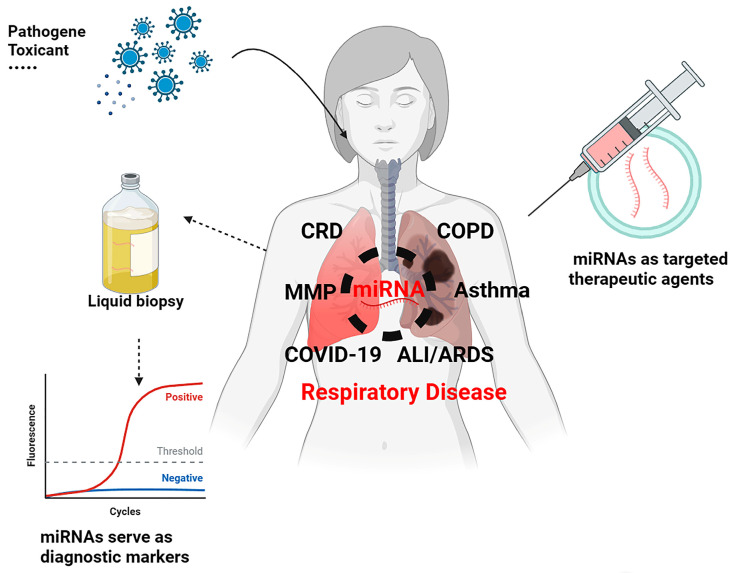
Graphical abstract. miRNAs possess the potential to serve as stable diagnostic biomarkers and therapeutic targets for several respiratory diseases.

In conclusion, our comprehensive analysis of miRNA dysregulation in Mycoplasma-induced respiratory diseases provides a robust foundation for the exploration of the translational potential of miRNA-based therapies in the broader landscape of respiratory diseases that share common pathogenic and regulatory mechanisms (Figure 5). By considering the role of miRNAs in diverse respiratory disease models, we identify conserved functions in regulating immune responses and inflammation. These conserved miRNAs, exemplified by miR-21, hold promise as therapeutic targets in managing immune dysfunction and inflammation in a range of respiratory diseases. Moreover, utilizing miRNAs such as miR-155 or miR-223 as diagnostic markers, while accounting for their distinct roles in different cell types, offers a path to early disease detection, prognosis improvement, and personalized therapies. To fully harness the potential of miRNA-based interventions, addressing disease-specific mechanisms and delivery strategies, while accounting for cell-specific responses, will accelerate the clinical translation of miRNA-based therapies in the realm of respiratory medicine.

## Figures and Tables

**Table 1:** Dysregulated miRNAs in MG infection.

miRNA	Target	Function	Reference
miR-451	YWHAZ	inhibits cell cycle progression and cell proliferation and promotes cell apoptosis	[28]
miR-193a	KRAS	inhibits cell proliferation, promotes apoptosis, and increases inflammation	[25]
miR-181a	PPM1B	activates the TLR2-mediated MyD88/NF-κB pathways to promote inflammation	[24]
miR-19a	ZMYND11	activates the NF-κB signaling pathway to defend against MG infection	[32]
miR-99a	SMARCA5	represses the proliferation of MG-infected DF-1 cells by inhibiting cell cycle	[22]
miR-101	EZH2	inhibits proliferation of MG-infected DF-1 cells by inhibiting cell cycle	[26]
miR-146c	MMP16	activates the TLR6/MyD88/NF-κB pathway to defend against MG infection	[30]
miR-16	PIK3R1	inhibits PI3K/Akt/NF-κB pathway to exert an anti-inflammatory effect	[35]
miR-130b	PTEN	activates the PI3K/AKT/NF-κB pathway	[31]
miR-21	MAP3K1	promotes inflammation and cell proliferation to defend against MG infection	[29]
miR-142	TAB2	facilitates cell proliferation by inhibiting cell apoptosis to defend against MG infection	[27]
miR-223	FOXO3	decreases proliferation and cycle progression, and increases apoptosis to promote MG infection	[33]
miR-24	RAP1B	decreases proliferation but increases apoptosis	[34]
let-7	MPK1	an inhibitor of MAPK pathway to effectively mitigate MG adhesion	[36]
miR-365	SOCS5	a key factor for MG evasion of host immunity	[20]
miR-33	JNK1	lnc90386 sponges miR-33-5p to defend against MG infection	[21]

**Table 2:** miRNAs in COVID-19/SARS-CoV-2.

miRNAs	Function	References
miR-1207	may contribute to uncontrolled inflammation in most severe COVID-19 cases	[55]
miR-21	has the largest probability of binding the human coronavirus RNAs	[56]
miR-146a miR-21 miR-142 miR-15b	as potential contributors to the disease pathogenesis, possibly serving as biomarkers of severe COVID-19	[57,58,59,60]
miR-126	involved in vascular endothelial function	[35]
miR-208a	associated with myocardial injury in COVID-19 patients	[60]
miR-200c let-7b	involved in the regulation of ACE2 expression	[61,62]
miR-223 miR-146a	enhance the antiviral immune response	[63,64]
miR-145 miR-885	may contribute to the thrombotic complications observed in COVID-19 patients	[65]
miR-148a miR-590	significantly upregulated in the exosomes of patients with neurological manifestations	[66]
miR-7 miR-24 miR-145 miR-223	are associated with high mortality rates of COVID-19 in the elderly	[67]
miR-588 miR-587 miR-582	enhance lung pathogenesis and injury	[68]

**Table 3:** miRNAs in COPD.

miRNAs	Function	References
miR-320d	suppresses inflammatory cytokine production by regulating NF-κB activity	[70]
miR-21	regulates the HIF-1α signaling pathway, which is responsible for myofibroblast differentiation, to treat COPD	[71]
let-7d miR-191	affect the clearance of apoptotic cells by specialized macrophages and may contribute to the pathogenesis of COPD	[72]
miR-206 miR-146a	have potential value for the diagnosis and treatment of COPD	[73,74]
miR-21 miR-181a	their ratio is suggested as a potential biomarker in predicting the development of COPD in asymptomatic smokers	[75]
miR-100 miR-20a miR-34c-5p miR-28-3p miR-7	potential biomarkers for COPD and may have a biological function in the pathogenesis of COPD	[76]
miR-452	increases the expression of MMP12 and causes emphysema	[77]
miR-638	positively correlated with emphysema severity	[78]
miR-199a-5p	decreases hypoxia inducible factor 1α (HIF-1α) expression	[79]

**Table 4:** miRNAs in ALI/ARDS.

miRNAs	Function	References
miR-214 miR-415 miR-16 miR-23a miR-24 miR-181 miR-181b miR-199a	a group of miRNAs that were differentially expressed in ALI mice; miR-16 may play a role in alleviating in ALI by inhibiting LPS-induced IL-6 and TNF-a	[86]
miR-29a	may play a role in regulating the inflammatory response in ALI by targeting TNFR1; reduces alveolar epithelial cell PANoptosis in the ALI mouse model	[87]
miR-27a miR-16 miR-182 miR-145-5p miR-140 miR-140-5p miR-146a	reduce the release of pro-inflammatory cytokines and downstream TLR4/MyD88/NF-κB signaling pathways, ultimately suppressing the inflammatory response	[88,89,90,91,92]
miR-146b	reduces lung inflammation and increases lung permeability by targeting IRAK1 to inhibit NF-κB signaling	[93]
miR-124-3p	promotes macrophage apoptosis and plays a protective role in ARDS by targeting p65	[94]
miR-21	inhibits the JAK2/STAT3 signaling pathway, thereby reducing the infiltration of inflammatory cells in the lung tissue of ALI/ARDS	[95]
miR-216a	inhibits the JAK2/STAT3 signaling pathway, inhibiting cell apoptosis, autophagy, and the release of inflammatory factors	[96]
miR-30b-5p	negatively regulates the JAK2/STAT3 pathway	[97]
miR-127	suppresses lung inflammation by targeting macrophage CD64	[98]
miR-92a	inhibits PI3K/AKT pathway, improves endothelial cell barrier function, and protects alveolar vascular endothelial cells	[99]
miR-21a-3p	regulates the PI3K (p110α)/Akt/mTOR pathway and promotes lung tissue repair and angiogenesis	[100]
miR-802	improves lung injury induced by LPS	[101]
miR-495 miR-223	inhibits NLRP3 activation, leading to reduced inflammation and improved ALI/ARDS	[102]

**Table 5:** miRNAs in asthma.

miRNAs	Function	References
miR-34a miR-206	modulate airway inflammation by regulating the expression of cytokines	[107,109]
miR-26	regulates airway remodeling by regulating the expression of collagen genes	[108]
miR-21	promotes airway remodeling by regulating the expression of matrix metalloproteinases	[110]
miR-155	promotes Th cell proliferation through downregulation of CTLA-4, thereby participating in the development of allergic asthma	[111]
miR-146a	reduces airway inflammation	[112]
let-7	reduces airway inflammation	[113]
miR-1248 miR-155 miR-26a miR-376a	potential biomarkers for asthma	[114,115,116]
let-7a miRNA-658 miRNA-24 miRNA-26a miRNA-99a miRNA-200c miRNA-1268	potential biomarkers and strongly correlated with forced expiratory volume in 1 s (FEV1) within asthmatic patients	[117]
miR-140	appears to play an influential role in airway smooth muscle cell hyperplasia	[118]
let-7 families miRNA-200 families	dysregulated in the exosomes isolated from BAL fluid of asthmatic patients, potential biomarkers for asthma	[119,120,121]
